# Response Inhibition During Differential Reinforcement of Low Rates (DRL) Schedules May Be Sensitive to Low-Level Polychlorinated Biphenyl, Methylmercury, and Lead Exposure in Children

**DOI:** 10.1289/ehp.9216

**Published:** 2006-08-18

**Authors:** Paul W. Stewart, David M. Sargent, Jacqueline Reihman, Brooks B. Gump, Edward Lonky, Thomas Darvill, Heraline Hicks, James Pagano

**Affiliations:** 1 Department of Psychology, State University of New York at Oswego, Oswego, New York, USA; 2 Agency for Toxic Substances and Disease Registry, Division of Environmental Medicine, Atlanta, Georgia, USA; 3 Environmental Research Center, State University of New York at Oswego, Oswego, New York, USA

**Keywords:** differential reinforcement of low rates, DRL, fixed interval, inhibition, PCBs, poly-chlorinated biphenyls

## Abstract

**Background:**

Animal studies have shown that exposure to common, low-level environmental contaminants [e.g., polychlorinated biphenyls (PCBs), lead] causes excessive and inappropriate responding on intermittent reinforcement schedules. The Differential Reinforcement of Low Rates task (DRL) has been shown to be especially sensitive to low-level PCB exposure in monkeys.

**Objectives:**

We investigated the relationships between prenatal PCB and postnatal Pb exposure performance on a DRL schedule in children. We predicted that *a*) prenatal PCB exposure would reduce interresponse times (IRTs) and reinforcements earned, and *b*) postnatal Pb exposure would reduce IRTs and reinforcements earned.

**Methods:**

We tested 167 children on a DRL20 (20 sec) reinforcement schedule, and recorded IRTs and the number of reinforced responses across the session. We measured prenatal PCB exposure (cord blood), methylmercury (MeHg) (maternal hair), and postnatal Pb exposure (venous blood), and > 50 potentially confounding variables.

**Results:**

Results indicated impaired performance in children exposed to PCBs, MeHg, and Pb. Children prenatally exposed to PCBs responded excessively, with significantly lower IRTs and fewer reinforcers earned across the session. In addition, exposure to either MeHg or Pb predicted statistically significant impairments of a similar magnitude to those for PCBs, and the associated impairments of all three contaminants (PCB, MeHg, and Pb) were statistically independent of one another.

**Conclusions:**

These results, taken with animal literature, argue the high sensitivity of DRL performance to low-level PCB, MeHg, and Pb exposure. Future research should employ behavioral tasks in children, such as DRL, that have been demonstrably sensitive to low-level PCB, MeHg, and Pb exposure in animals.

Research examining the impact of low-level polychlorinated biphenyl (PCB) exposure on the behavior of children has been performed in a variety of studies over the past several years (Jacobson and Jacobson 1996, 2003; Patandin et al. 1999; Stewart et al. 2005; Walkowiak et al. 2001). In general, the data have supported the hypothesis that low-level PCB exposure predicts small but statistically significant impairments on global tests of cognitive function (Jacobson and Jacobson 1996; Patandin et al. 1999; Schantz et al. 2003; Stewart et al. 2003a; Walkowiak et al. 2001), although an exception has been noted (Gray et al. 2005). Regardless of the merits of the evidence concerning the impact of PCB exposure on global cognitive development in children, one cannot help but be struck by the disconnect between the approaches to studying the neurobehavioral associations with PCBs in humans versus the approaches to studying these same effects in animals. The child cognitive development literature is generally dominated by reliance on standardized, psychometric assessments of global cognitive development, which rely heavily on language for proper performance. In contrast, research with laboratory animals has often involved assessments of specific behavioral domains that require classical experimental approaches. These investigations include the study of PCB-related effects on schedule-controlled behavior (Berger et al. 2001; Lilienthal et al. 1990; Rice 1997, 1999), as well as the assessment of several domains of nonverbal learning, such as attention and associative processing (Widholm et al. 2004), spatial learning (Widholm et al. 2001), and behavioral inhibition (Rice 1997, 1998, 1999).

In several important ways, animal and human research can perform complementary functions to cross-validate the associations seen in the respective literatures. In most cases, however, human research can benefit the most from this relationship: It is far easier to adapt tests used to evaluate neurobehavioral functions from animals to humans than the reverse. Even further, significant PCB findings from carefully controlled, animal experiments usually leave little doubt that PCBs are the causative agent, rather than some unknown confound. Thus, animal results can provide strong evidence about the specific neurobehavioral functions that are impaired by PCB exposure, and direct us toward the specific measures most sensitive in detecting these effects.

Growing evidence in the animal literature demonstrates that behavioral inhibition during reinforcement schedules is quite sensitive to several environmental contaminants, including PCBs (Berger et al. 2001; Lilienthal et al. 1990; Rice 1997, 1998, 1999) and post-natal lead (Cory-Slechta 1990; Cory-Slechta et al. 2002). Nowhere is this more clearly demonstrated than in studies of the performance of animals on delayed reinforcement paradigms. These are schedules where there are delays between the reinforcers and the responses, whether these delays are contingent on the animal’s behavior or not.

Studies of performance on Fixed-Interval (FI) and Differential Reinforcement of Low Rates (DRL) schedules provide two examples of these approaches. Both FI and DRL schedules employ fixed intervals of time (delays) that must expire before a given response is reinforced. For example, in an FI20 or a DRL20 schedule, a response will not be reinforced until at least 20 sec have elapsed since the previously reinforced response. The difference between these schedules is that responses before the expiration of the interval during FI schedules are without consequence, whereas making such premature responses on DRL schedules resets the interval clock to the beginning, resulting in delay of reinforcement. Despite these differences, both these schedules have in common the requirement to learn to withhold responses before termination of the delay interval. Such behavioral performance can rightly be characterized as a form of response inhibition (Barkley 1997, 1999).

Interestingly, exposure to both postnatal Pb and postnatal PCBs cause excessive and premature responding on both FI and DRL schedules in animals. Similarly, several well-conducted studies have demonstrated that low-level postnatal Pb exposure causes rats to respond inefficiently and with greater frequency on FI and DRL schedules, as evidenced by an increase in the proportion of short inter-response times (IRTs) on FI schedules (Cory-Slechta et al. 2002) and shorter IRTs and fewer reinforcements on DRL schedules (Rice 1992). In the case of PCB exposure, monkeys exposed to an environmental-relevant mixture of PCBs, at levels found within human populations, showed a much greater response rate during the fixed interval (i.e., shorter IRTs) and, consequently, retarded acquisition of the schedule (Rice 1998). This finding has been replicated with rats (Berger et al. 2001; Carpenter et al. 2002). Rice (1998) also reported a large and statistically robust impairment on DRL schedules in monkeys exposed to low levels of PCBs. These PCB-exposed primates responded excessively and inefficiently, resulting in fewer earned reinforcements. Rice (1998) described the effects of PCBs as an inability to withhold or delay inappropriate responding.

These data provide important clues about the behaviors and assessment tools that would be most sensitive to PCB exposure in humans. Yet it is unfortunate that not a single study has attempted to replicate these findings in children exposed to PCBs or Pb, let alone dissociate such putative effects from confounding contaminants, such as methylmercury (MeHg). This shortcoming is further underscored by the observation that there already exist clues in the literature on humans to suggest that exposed children will exhibit similar response inhibition impairments to those observed in animals. Stewart et al. (2003b) showed that children exposed prenatally to PCBs have demonstrated excessive responding during continuous performance tasks (CPT), which are traditional measures of attention and impulse control. A follow-up study suggested these deficits appear to be caused not by a tendency to initiate large numbers of responses, but rather by an impairment in the ability to inhibit and/or withhold them (Stewart et al. 2005).

Given the wealth of animal data showing PCB- and Pb-related impairments on delayed reinforcement schedules on one hand and the emergent human studies of impulsive behavior on the other (Stewart et al. 2003b, 2005), we examined performance on delayed reinforcement schedules in children exposed to environmental mixtures of PCBs, MeHg, and Pb. Specifically, we examined performance of exposed children on a DRL schedule of reinforcement, using protocols very similar to primate studies in which low-level PCB and Pb effects on DRL performance were found (Rice 1992, 1997). We also chose DRL because, unlike with FI schedules, impulsive responding on DRL schedules can have a direct consequence of reducing the rate of reinforcement. This gives substantive significance to DRL impairments because a functional consequence to impaired behavior can be demonstrated. Our predictions in this study were derived directly from the animal literature. Specifically, we predicted that *a*) prenatal PCB exposure would reduce IRTs and reduce reinforcements earned (Berger et al. 2001; Rice 1998), and *b*) postnatal Pb exposure would reduce IRTs and reinforcements earned (Cory-Slechta et al. 2002; Rice 1992).

## Methods

### Subjects

Participant mothers and their children currently enrolled in the Oswego Study (*n* = 202) participated in this study as part of an ongoing, longitudinal study of the relationship between prenatal PCB exposure and cognitive development in children. The sampling methodology and demographic and exposure characteristics of this cohort have been previously published in detail (Lonky et al. 1996; Stewart et al. 1999, 2000a, 2000b). Of the 202 children available for testing, 195 had valid PCB exposure data, and 183 of these children completed the DRL task at 9.5 years of age. Data transfer (save) failure occurred in 16 subjects, resulting in available data for 167 PCB-exposed participants. Children who did not complete the DRL task were typically unavailable because of a parent’s scheduling conflicts during the testing window. Recent analysis of subject attrition revealed no participation bias with respect to contaminant exposure (Stewart et al. 2003a). Institutional review board approval regarding the nature and specifics of the research was obtained before testing with any subjects, and all participants signed written informed consent that was collected by behavioral assessors before testing began.

### Classification of exposure

Immediately after birth, a sample of umbilical cord blood was obtained for analysis of PCBs, dichloro-diphenyldichloroethylene (DDE), and hexa-chlorobenzene (HCB) by capillary column gas chromatography (mean cord total PCB = 0.96 ng/g; DDE = 0.15 ng/g; HCB = 0.071 ng/g). Sample collection and analytic methods have been described elsewhere (Stewart et al. 1999). Prenatal PCB exposure was assessed previously in terms of total cord PCBs (the sum of all 68 congeners and coeluters) and the sum of highly chlorinated PCB congeners (hepta-, octa-, and nonachlorinated biphenyl homologues; see Stewart et al. 1999, 2000a).

Assessment of heavy metal exposure included prenatal MeHg, prenatal Pb, and postnatal Pb. For prenatal MeHg, 5-cm maternal hair samples were collected within 24 hr of birth for MeHg analysis at the University of Rochester (mean hair MeHg = 0.56 μg/g). Prenatal cord Pb levels (mean cord Pb = 1.81 μg/dL) were measured from cord blood venipuncture and analyzed at the New York State Department of Health (Gump et al. 2005). Unlike the previously mentioned contaminants, postnatal Pb levels (mean postnatal Pb = 4.58 μg/dL) were collected from the medical records of each child’s physician. Because postnatal Pb levels were collected through state-mandated testing at each physician’s office, postnatal Pb levels were collected using both capillary samples (20.1%) and venipuncture (79.9%). However, blood collection method was unrelated to Pb level. Postnatal Pb was measured between 2 and 4 years of age (median age = 2.6 years). The sample collection and analytic methods for postnatal Pb have been previously described (Gump et al. 2006).

### Apparatus

**Figure 1:**
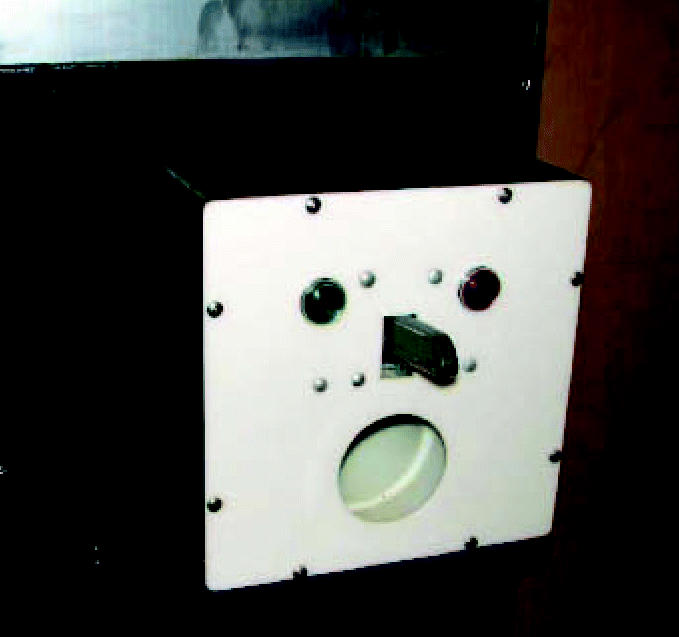
Operant panel for children, based on Sagvolden et al. (1998).

The operant panel (response manipulanda and reinforcer delivery mechanism) was built in the form of a clown face (Figure 1) based on a similar device by Sagvolden et al. (1998). The operant panel was affixed to a standard electromechanical equipment rack (192 cm high by 50 cm wide). The clown face was built on a 25-cm square piece of white opaque plexiglass attached to a black painted wooden cube 25 cm on a side. This cube was mounted on the electromechanical rack 81 cm above the floor. The eyes of the clown face were 28VDC 10-watt stimulus lights with 2.75-cm jewel lenses mounted 10 cm apart on center and 19 cm above the bottom of the plexiglass panel. The reinforcement delivery aperture was a 7.5-cm hole 4.5 cm from the bottom of the panel forming the mouth of the clown face. The response lever was made from a standard utility knife handle (without blade) with a spring pivot mechanism serving as the nose of the face. The knife handle was connected to a microswitch providing an activation force of 570 g when combined with the spring.

On the back side of the equipment rack, a conveyor belt driven by a stepper motor was used to deliver marbles to a feeder tube connected to the reinforcement aperature (clown’s mouth). The stepper motor, stimulus lights, and response microswitch were all connected to standard 28VDC electromechanical relays and pulse formers. The 28VDC equipment was connected to a standard microcomputer through a computer bus interface system. Specifics on the electrical and electronic connections and equipment can be found in previous publications (Sargent 1989, 1991; Sargent and Deelman 1995).

The DRL program, along with the controls for all the electrical equipment, was written into a BASIC program (QuickBasic, Microsoft, Redmond, WA). The program delivered a marble for each of the first 10 presses of the knife handle lever followed by a DRL20 schedule. If the lever was pressed before the completion of 20 sec, the time was reset to 20 sec and no marble was delivered. The program sequentially recorded the IRT of each lever press to within a hundredth of a second. The sequential IRTs were stored in the computer for analysis.

### DRL testing protocol

All testing was conducted in our laboratory at the Psychology Department at the State University of New York at Oswego. At the start of the activity, the subjects were asked to sit in a chair approximately 1 foot away from the covered DRL machine. The behavioral assessor then set up the task on the computer and addressed the subject. The child is told that he or she will be read some instructions, which is done so that every child learns how to play the game the exact same way. The instructions, based on the work of Sagvolden et al. (1998), were then read to the subject: “Now before we begin, this game has magnets in it, so you need to remove any watches, rings, or bracelets you have on.” (The behavioral assessor pauses to give the subject a chance to remove any jewelry.) “You are going to play a game in which you will win some marbles that you can trade in for money. You can win 5 cents for every marble you earn, but you have to earn them on your own. That means I can’t talk to you while you’re playing the game. It is fine if you speak to me while you are playing, but I can’t answer any questions. I will tell you when the game starts, and when it’s over. Here is the game [behavioral assessor uncovers the DRL machine, bearing resemblance to a clown face] and here is the cup that you have to put your marbles in [behavioral assessor hands subject a round plastic dish]. You have to remove each marble as it drops and put it in your cup—you can’t let the marbles build up in the clown’s mouth [assessor points to the ‘mouth’ of the machine, where marbles are dispensed into]. You can start any time you are ready.”

At this point, the assessor started the program, which started the timer and turned the lights on in the machine. Throughout the activity, the assessor sat behind the machine, out of view of the subject, monitoring the length of time the child had played and number of marbles earned. If, after 15 min, the child had not yet pulled the lever even once, the assessor prompted the subject with a cue: “Now remember, the game doesn’t run itself —you have to run it. That means you have to touch things on the game and physically play with the game in order to get it to work. Otherwise, the game will not do anything, and you won’t get any marbles.”

The task was permitted to last no more than 1 hr. As a general rule, the behavioral assessors did not address the subjects or engage in conversation during the activity; however, there were certain exceptions. If children hit the machine excessively, they were asked to please not do so, and the behavior was noted. Children were also asked to return to their seats if they were engaging in disruptive behaviors. After the activity, children were rewarded 5 cents for every marble they earned.

### Statistical methodology

#### Measurement of potential confounders

We collected data for potential confounding variables from neuropsychological testing instruments, standardized psychometric test batteries, hospital records, structured interviews, and repeated assessments of the home environment (HOME; home observation for measurement of the environment) and socioeconomic status (SES) (Bradley et al. 1992). Several measures of maternal intelligence and neuropsychological performance were measured in this study. Maternal IQ was assessed twice, using the Peabody Picture Vocabulary Test (PPVT) (Dunn 1981) and the Kaufman Brief Intelligence Test (K-BIT) (Kaufman 1990). The correlation between these two IQ tests was *r* = 0.71, *p* < 0.001, so the average of the two IQ tests was used as the maternal IQ metric. Maternal Color-Word Interference (a measure of cognitive interference control) was also assessed using the Neurobehavioral Evaluation System 2 (NES2) (Letz 1988). Maternal sustained attention and impulsive responding were measured through the use of a Continuous Performance Test program (Stewart et al. 2005). Hollingshead SES data were updated with the Hollingshead 4-factor socioeconomic scale (Hollingshead AB, unpublished data). Details regarding the collection of demographic and all other covariate data are described elsewhere (Lonky et al. 1996; Stewart et al. 2000b). A small number of data could not be obtained for maternal sustained attention and the updated (9-year) SES measure in 3.9% and 8.6% of the mothers, respectively. These data were missing due to either computer malfunction or scheduling conflicts with the mother. In the case of SES update at year 9, the low percentage of missing covariate data was estimated with reasonable accuracy using a multiple stepwise regression with previous Hollingshead SES, the latest HOME measure of the environment, and maternal education level (multiple *r* = 0.67, *p* < 0.0001). For maternal sustained attention, we predicted missing covariate data using maternal IQ, the latest HOME score, and Maternal Color-Word Interference scores (multiple *r* = 0.35, *p* < 0.0001). Importantly, all outcomes reported in this article (see “Results”) remained significant whether or not these subjects were excluded or included in the analysis. A list of all the covariates considered in the present analysis, and their correlations with outcome measures, is shown in Table 1.

#### Statistical treatment of potential confounders

The decision rules for the inclusion of covariates provide for objective, comprehensive, and rigorous control for potential confounders. The method used in the current study is consistent with that employed previously (Darvill et al. 2000; Stewart et al. 2000b, 2003a, 2003b, 2005), as well as others (Jacobson and Jacobson 1996). Any potential confounding variables even marginally related (*p* < 0.20) to performance served as covariates in all analyses. Several studies (e.g., Maldano and Greenland 1993; Mickey and Greenland 1989) have demonstrated this alpha level (0.20) is effective at guarding against confounders in Monte Carlo simulations. This criterion also allows major predictors of outcome to enter the equation, even if these variables are unrelated to exposure. Statistical power is thereby increased by reducing the error term in the regression equation (Kleinbaum et al. 1988). Relationships between covariates and outcome were determined through single-pass, bivariate correlations between each covariate and outcome.

To further minimize residual confounding, we tested each covariate failing to meet the *p* < 0.20 entry criterion (above) to see if it affected the final outcome of the analysis. Monte Carlo simulations have empirically demonstrated that this additional change-in-estimate criterion, whereby a covariate is added to the equation if it changes the association (beta coefficient) between exposure and outcome by ≥ 10%, is a quite effective and rigorous means of controlling residual bias in multivariate correlational datasets (Maldano and Greenland 1993; Mickey and Greenland 1989). We included every single covariate that even marginally (> 10%) altered the coefficient associated with exposure. We are afforded strong assurance that no potentially important confounder was excluded. This approach has been used successfully in the literature on Pb (Bellinger et al. 1992) and PCBs (Stewart et al. 2005).

Every analysis, therefore, included all covariates related to the outcome at *p* < 0.20, and any remaining covariates that even marginally changed the relationship between exposure and outcome (> 10% change in beta). Only relationships that were statistically significant after this two-tiered approach were considered meaningful.

#### Statistical treatment of the predictor variable (PCBs)

We assessed the associations of total PCB, DDE, HCB, MeHg, and Pb exposure with DRL performance parameters using a linear regression model. In some cases, significant findings with regression were followed by dose–response analyses by creating dose groups (i.e., quartiles). Because highly chlorinated PCBs were measured in an ordinal fashion (Stewart et al. 2001), the associations with this variable were assessed using analysis of covariance (ANCOVA) across four dose groups with a linear *F*-test, as described previously (Stewart et al. 2005, 2003a, 2003b). Whether regression or ANCOVA was employed, all covariates meeting the criteria for entry were entered in the first step, followed by the specific contaminant in the second step. If any contaminant other than the one under consideration met the criterion for a possible confounder, it also served as a covariate in the analysis. For example, significant associations with PCBs controlled for associations with MeHg, and vice versa. Potential interactions among PCB, MeHG, and Pb were also assessed. Two-tailed significance tests (alpha = 0.05) were used in all analyses.

### DRL performance parameters

Unlike animal studies, where mastery of the schedule can take days or weeks (Berger et al. 2001), there were practical and ethical limits to what could be asked of the children enrolled in this study, and a 1-hr limit was established for each child. Thus, the present article is more properly an analysis of the acquisition of a DRL schedule within a single session, an approach well suited to children because most can learn the DRL schedule within the session.

We analyzed the acquisition of DRL performance across the training session by examining the IRTs in each of a series of response bins (i.e., bins of 20 responses each). We employed this approach to control for response history. Learning during a DRL schedule is a result of feedback from one’s cumulative responses, not from the amount of time that passes during the session. By comparing the performance (IRTs) among each subject at controlled points in their response history, we can assess the learning of the schedule without confounding by interindividual differences in reinforcement history and experience with the schedule.

Because a single IRT was associated with each response, and each subject generated well over 100 IRTs, we analyzed IRTs for each subject in sequential bins of 20 responses each. Because the distributions of IRTs within each bin often demonstrated extreme positive skew, we used the median IRT for each subject to reduce the influence of outlier IRTs. Complete data for every subject were available for a total of seven response bins (7 bins × 20 responses each = 140 total responses). Missing data grew rapidly beyond the seventh response bin, because by this time, the 1-hr test limit was reached for almost all subjects who spaced their responses carefully (20–30s IRT) across the testing session. Thus, for analysis purposes we analyzed the complete data set of seven response bins to examine within-session learning, whereas we analyzed the mean performance averaged across all response bins to examine overall performance. Further, the cumulative number of nickels earned was recorded to measure the total number of reinforced responses.

## Results

**Figure 2:**
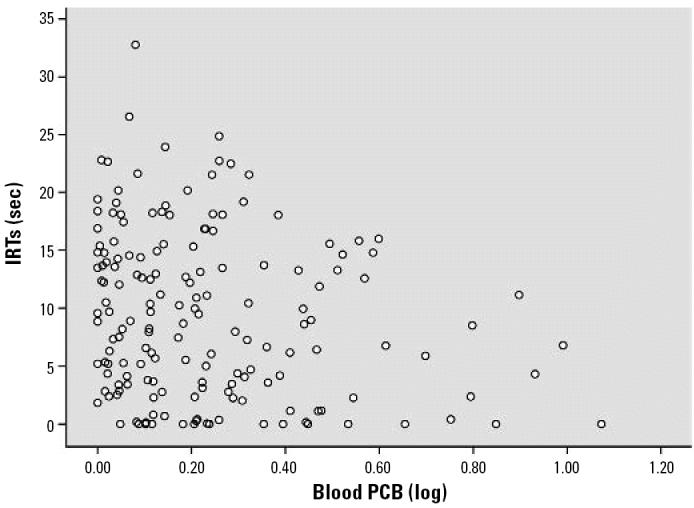
Scatterplot of the covariate-controlled relationship between total cord blood PCBs (log x +1) and the IRT of each subject. A significant negative relationship (β = −0.215, *p* = 0.008) is observed.

**Figure 3:**
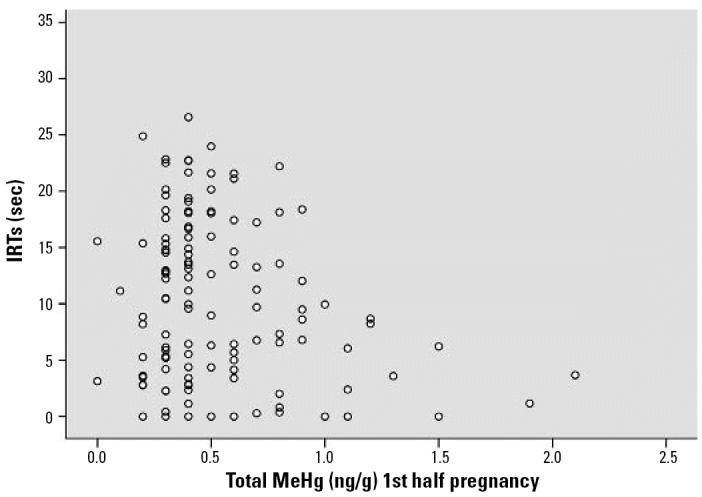
Scatterplot of the covariate-controlled relationship between total maternal hair MeHg, taken during the 1st half of pregnancy, and the IRT of each subject. A significant negative relationship (β = −0.179, *p* = 0.040) is observed.

**Figure 4:**
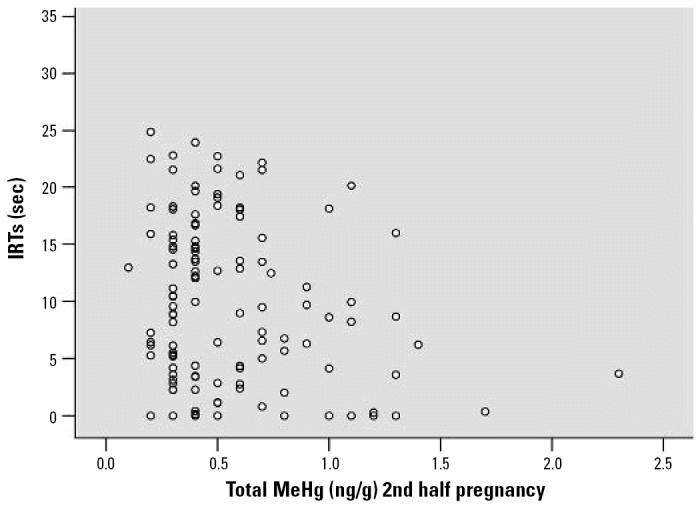
Scatterplot of the covariate-controlled relationship between total maternal hair MeHg, taken during the 2nd half of pregnancy, and the IRT of each subject. A significant negative relationship (β = −0.219, *p* = 0.015) is observed. Exclusion of the single subject with the MeHg level > 2.0 did not change the results (β = −0.192, *p* = 0.034 with subject removed).

**Figure 5:**
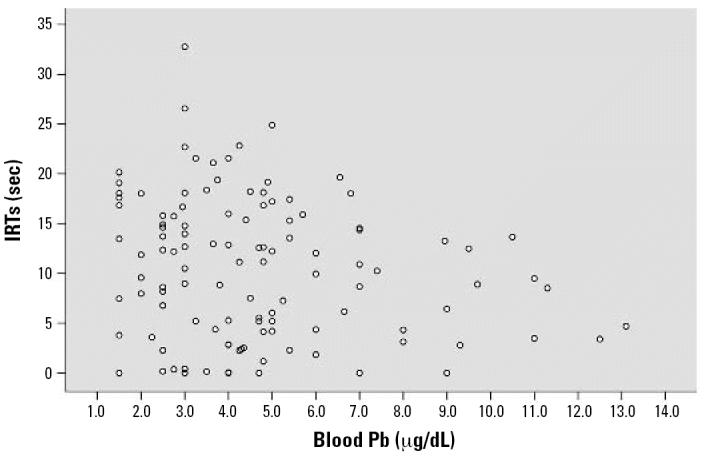
Scatterplot of the covariate-controlled relationship between post-natal blood Pb (obtained at 2.6 years of age) and the IRT of each subject. A trend for a negative relationship (β = −0.194, *p* = 0.081) is observed.

The covariate-controlled relationships of PCB, MeHg, DDE, HCB, and Pb exposure with DRL performance are shown in Table 2 and Table 3. Inferential statistics are expressed in standardized regression coefficients (betas). IRTs increased across the testing session (time *F* = 33.09, *p* < 0.001). Both total and highly chlorinated PCBs were associated with shorter IRTs [total PCB β = −0.215, *p* = 0.008; highly chlorinated PCB linear *F* = 4.79, *p* = 0.030 (β = −0.170, *p* = 0.030)], and total PCB was associated with fewer reinforcements earned (β = −0.208, *p* = 0.010). Prenatal MeHg exposure in both the first and second half of pregnancy was associated with shorter IRTs (MeHg first half of pregnancy β = −0.179, *p* = 0.040; MeHg second half of pregnancy β = −0.219, *p* = 0.015; combined MeHg β = −0.178, *p* = 0.024). Both MeHg metrics were associated with fewer reinforcements earned (MeHg first half pregnancy β = −0.194, *p* = 0.026; MeHg second half pregnancy β = −0.203, *p* = 0.027). Postnatal Pb exposure showed a relationship trend with shorter IRTs (β = −0.177, *p* = 0.081) and a significant association with fewer reinforcements earned (β = −0.195, *p* = 0.047). The remaining contaminants (DDE, HCB, and prenatal Pb) were not associated with DRL performance. No interactions were observed among any contaminants (all *p*-values > 0.10). Figures 2–5 show the scatterplots of the relationships among PCB, MeHg (first half of pregnancy), MeHg (second half of pregnancy), postnatal Pb, and IRTs. PCBs, MeHg, and Pb revealed a simlar pattern of associations. Higher exposures were associated with both shorter IRTs and less between-subject variability. Generally speaking, almost every subject who fell within the top 10% of exposure to any of the three contaminants did poorly on the DRL test.

The presence of associated impairments across three contaminants underscores the need to parcel out the associations of each contaminant from the other. Results of this approach revealed that the associations of each contaminant were clearly independent of one another. Table 4 illustrates the beta coefficients for PCBs and MeHg, with and without control for the other. The number of subjects for whom both PCB and MeHg were available was smaller (*n* = 145) than the number of subjects with either contaminant alone. Nevertheless, both the PCB and MeHg associations were significant in the subsample, and both remained significant after control for the other.

A considerably smaller sample size in the postnatal Pb subsample made the multivariate assessments with PCBs and MeHg more challenging. The number of subjects with valid Pb and PCB data was 115. The number of subjects with valid Pb and MeHg data was 105. With such a marked drop in sample size and statistical power, neither the PCB effect (β = −0.215) nor the MeHg effect (β = −0.178) could attain significance in the Pb subsample, even before Pb was applied as a covariate. This precludes the ability to test the ability of PCB and MeHg associations to withstand application of Pb as a covariate. However, Pb levels were quite unrelated to total PCB (*r* = 0.00, *p* = 0.99), highly chlorinated PCB (*r* = 0.02, *p* = 0.80), and MeHg (r = −0.06, *p* = 0.54). Such observations make it very improbable that either the PCB or MeHg associations are attributed to confounding with Pb, or vice versa.

## Discussion

The results of this study support the hypothesis that performance on delayed reinforcement paradigms—in this case DRL—is highly sensitive to PCBs, Pb, and, unexpectedly, MeHg. Impaired DRL performance, whether indexed through reduced IRTs or fewer reinforcements earned, was significantly associated with pre-natal PCB exposure, prenatal MeHg exposure, and postnatal Pb exposure. Each of these associations was statistically independent of the others. The outcomes for PCBs and Pb in this study were almost exactly as predicted based on the empirical and theoretically based predictions. The PCB and Pb associations were specifically predicted based on an extant animal literature (Cory-Slechta et al. 2002; Rice 1992, 1997, 1999; Widholm et al. 2001, 2004). The animal literature concerning both PCBs and Pb has repeatedly demonstrated excessive responding on delayed or intermittent reinforcement schedules in exposed animals (Cory-Slechta 1990; Lilienthal et al. 1990; Rice 1992, 1997, 19992). Previous reports from our research group have provided compelling evidence of a pattern of impaired response inhibition in children exposed to PCBs and, to a lesser extent Pb (Stewart et al. 2003b, 2005b). Finally, impaired response inhibition and excessive responding during delayed reinforcement paradigms is consistent with alterations in the function of the pre-frontal cortex (PFC). Early observations by Levin (1992) suggested a pattern of behavior in animals exposed to Pb and PCBs consistent with PFC dysfunction.

One potential criticism of the current study is that the median IRTs of many children did not exceed the 20-sec reinforcement threshold. This may raise the question of whether all the children learned the task. This concern rests on a false assumption that the median IRT must exceed the reinforcement criterion for the DRL schedule to be learned. The median IRT for each subject is by definition the 50th percentile, with half of the remaining IRTs falling above and half below this value. Clearly, even if a subject’s median IRT met the criterion of 20 sec, half of his or her responses would still fall below that criterion. Thus, although it is important to examine IRTs to examine temporal response characteristics, we also examined the percentage of reinforced responses. This latter value provides direct information concerning the percentage of responses that met the DRL criterion. The percentage of reinforced responses increased across the session in all the groups, a pattern indicative of learning. Further, all three contaminants—PCBs, Pb, and MeHg—were associated with a reduced percentage of reinforced responses. This indicates retarded acquisition of the DRL schedule. Nearly all children evidenced various degrees of learning of the DRL task, and PCBs, Pb, and MeHg were associated with impairments in that process.

In comparing the relative effect sizes of PCBs, MeHg, and Pb, it is instructive to examine the beta coefficients and not just the *p*-values. If one relied on *p*-values alone, one might conclude that the major associations in the current study were found principally with PCBs and MeHg, but less so Pb. The former two contaminants (PCBs and MeHg) showed significant relationships with IRTs and reinforcements, whereas the latter (Pb) showed a statistical trend (*p* = 0.08) toward impairment. Yet the beta coefficient for postnatal Pb (β = −0.194) and IRTs was essentially the same as, and in some cases higher than, those seen for PCBs and MeHg (β = −0.155 to β = −0.215). It is quite possible that the smaller number of subjects with available postnatal Pb levels (*n* = 123) precluded an effect size that was identical to those of PCBs and MeHg from reaching conventional significance. Further, the statistically significant reductions in reinforcements earned in Pb-exposed children argues that Pb was a significant predictor of at least one of the two performance parameters. Put another way, this study shows remarkably consistent effect sizes across PCBs, MeHg, and Pb. The associated *p*-values for postnatal Pb on IRT performance may be weaker principally because of smaller sample size.

A few issues concerning the nature of the reported associations should be discussed. First, if postnatal Pb predicted performance impairments, why did prenatal Pb not do the same? And second, what implications does the additivity between PCB and MeHg in this study have for other data that show interactivity between PCBs and MeHg? (Bemis and Seegal, 1999, 2000; Stewart et al. 2003a). In regard to the first question, we specifically predicted that postnatal Pb would predict impaired performance because a large volume of animal literature relating Pb to impulsive operant responding is found with postnatal exposures (Brockel and Cory-Slechta 1998; Cory-Slechta 1990; Cory-Slechta et al. 1996, 1998). We are not aware of any literature of comparable volume that relates prenatal Pb to impulsive responding on operant schedules. In reference to the second question, it is true that there is some evidence that PCBs and MeHg interact to predict changes on biological (Bemis and Seegal 1999, 2000) and behavioral (Stewart et al. 2003a) end points. However, the fact that PCBs and MeHg may interact to affect some mechanisms does not mean that one should expect to find that PCBs and MeHg always interact on every behavior or mechanism that is examined. Indeed, Widholm et al. (2004) showed PCB and MeHg effects on learning in rats that were independent and nonadditive. Neurobehavior is not the product of a single mechansim, nor should we expect that PCBs and MeHg always work through the same mechanism(s). The understanding of the mechanisms of Pb and MeHg is far from complete, as is the understanding of brain–behavior mechanisms. Given these observations, the current findings should not be viewed as contradictory to evidence of PCB × MeHg interactions. In fact, additivity on some end points plus interactive effects on others are probably more likely than either alone.

Whatever the mechanism(s) of effect, if we momentarily assume that the current associations are causative, then the mechanisms are extraordinarily sensitive to exposure. The sensitivity of DRL to the neurotoxicity of environmental contaminants is underscored by the very low exposure levels in this cohort. The PCB exposure levels in this study are approximately one-third those found in the Michigan cohort, and far less than the exposure levels found in the Faroe Islands, German, or Dutch cohorts (Longnecker et al. 2003). The MeHg levels are near background (0.5 μg/g maternal hair), several orders of magnitude lower than the Faroe (Grandjean et al. 1992) and Seychelle (Cernichiari et al. 1995) Island cohorts. Finally, postnatal Pb levels average 4.58 μg/dL in venous blood, far below the level of concern of 10 μg/dL set by the Centers for Disease Control and Prevention (1991). That three unrelated contaminants, all at very low levels, predict impaired DRL performance is compelling evidence for the sensitivity of this task. Taken together, the evidence concerning the sensitivity of delayed reinforcement paradigms to environmental contaminants in general, and the evidence for these associations in this study in particular, is compelling.

## Correction

In Table 1, some of the SES correlations for 1 year and 9 years were incorrectly noted as negative in the original manuscript published online. All scores were positive, and have been corrected here.

## Figures and Tables

**Table 1:** Relationships between covariates and DRL performance (IRTs).

	Response bin^a^						
Covariates	1–20	21–40	41–60	61–80	81–100	101–120	121–140
Demographic							
Maternal education	−0.030	0.061	0.136*	0.151**	0.183*	0.104*	0.081
Paternal education	−0.030	0.093	0.161**	0.177**	0.234#	0.162**	0.136*
Parity of child	0.066	0.064	0.143*	0.061	0.061	0.056	0.064
SES score (1 year)	0.035	0.021	0.122*	0.144*	0.189**	0.136*	0.095
SES score (9 year)	0.027	0.140*	0.073	0.041	0.020	0.030	0.013
Maternal IQ [(PPVT + K-BIT)/2]	−0.002	0.028	0.109*	0.029	0.124*	0.142*	0.139*
Maternal sustained attention (CPT)	0.025	0.036	0.010	−0.010	0.061	0.034	0.072
Maternal impulsive responding (CPT)	−0.082	−0.027	−0.014	−0.076	−0.040	−0.032	−0.052
Maternal color-word vigilance (NES2)	0.003	0.016	0.011	−0.034	−0.074	−0.051	−0.014
Maternal depression (historical)	0.106*	0.196*	0.188**	0.077	0.049	0.063	0.073
Maternal depression (current)	0.085	0.040	0.047	0.060	0.043	0.065	0.064
Maternal age	0.027	0.045	0.151**	0.116*	0.168**	0.098*	0.085
Maternal height	−0.016	0.024	0.038	0.061	0.100*	0.115*	0.112*
Paternal age	−0.005	−0.004	0.123*	0.120*	0.162**	0.086	0.084
Paternal height	0.001	−0.066	−0.030	0.019	0.032	−0.008	0.025
Paternal weight	0.056	0.029	0.075	0.059	0.062	0.025	0.016
HOME 1 year	−0.033	−0.065	−0.007	0.066	0.101*	0.046	0.014
HOME 4.5 years	−0.090	−0.068	0.017	0.058	0.134	0.088	0.118*
HOME 7 years	−0.103*	−0.071	−0.016	0.044	0.090	0.068	0.063
No. of years at same address	−0.014	0.051	0.037	0.008	0.071	0.084	0.087
No. of years near Great Lakes	0.101*	0.092	0.188**	0.172	0.166**	0.129*	0.089
Marital status	0.066	0.069	−0.008	0.051	−0.027	−0.001	0.005
Day care facility	0.043	0.064	0.103*	0.129*	0.151**	0.111*	0.061
Home care	−0.077	−0.108*	−0.126*	−0.118*	−0.105*	−0.101*	−0.113*
Health/nutrition							
Prepregnancy weight	−0.007	0.011	−0.074	−0.064	−0.098*	0.003	−0.122*
Weight gain during pregnancy	−0.131*	0.008	−0.021	0.013	0.011	−0.012	0.067
Stress before pregnancy	−0.033	−0.003	0.048	0.057	0.071	0.055	−0.014
Stress: 1st half of pregnancy	0.062	0.023	0.054	0.003	0.006	0.017	0.014
Stress: 2nd half of pregnancy	0.027	0.059	0.101*	0.082	0.116*	0.085	0.079
Maternal illness history	−0.060	−0.008	0.002	−0.040	−0.057	−0.065	−0.079
Obstetric optimality	0.018	−0.044	−0.012	−0.035	0.037	−0.019	0.015
Vitamins during pregnancy	0.063	0.008	0.016	0.068	0.056	0.039	0.052
Prescription meds during pregnancy	−0.090	−0.074	−0.063	−0.131*	−0.128*	−0.092	−0.150**
Nonprescription meds pregnancy	−0.165**	−0.011	−0.004	−0.009	−0.016	−0.001	−0.012
Nutrition scale	−0.043	−0.047	−0.065	−0.059	−0.040	0.031	−0.008
Infant/birth characteristics							
Child sex	0.179**	0.132*	0.118*	0.172**	0.160**	0.171**	0.064
Birth weight (g)	−0.002	0.040	0.090	0.019	0.066	0.044	0.075
Head circumference (in)	−0.083	−0.054	−0.034	−0.076	−0.020	−0.031	−0.007
Ballard: neuromuscular^b^	−0.070	0.029	−0.015	0.017	0.022	−0.006	−0.011
Ballard: physical^b^	0.010	0.123*	0.108*	0.028	0.080	0.044	0.077
Gestational age at birth (weeks)	0.075	0.168**	0.152**	0.141*	0.162**	0.113*	0.088
Erythrocyte porphyrin (cord)	−0.104*	−0.018	−0.047	−0.091	−0.097*	−0.110*	−0.034
Substance use							
Cigarettes/day	−0.041	−0.076	−0.068	−0.065	−0.100*	−0.117*	−0.103*
Secondhand smoke (hr/day)	−0.035	−0.094	−0.162**	−0.182**	−0.236#	−0.220#	−0.243#
Alcohol (drinks/day)	−0.052	−0.007	−0.015	−0.030	−0.065	−0.090	−0.047
Herbal tea (drinks/month)	−0.011	0.030	0.018	0.066	0.083	0.095	0.017
Decaffeinated coffee (drinks/month)	−0.033	0.003	0.063	0.113*	0.145*	0.106*	0.081
Diet soda (drinks/month)	0.111*	0.044	0.132*	0.187#	0.233**	0.250#	0.127*
Decaffeinated soda (drinks/month)	0.029	0.032	0.100*	0.081	0.102*	0.143*	0.048
Caffeinated beverages (drinks/month)	−0.037	−0.049	−0.081	−0.113*	−0.091	−0.108*	−0.102*

a Twenty responses in each bin.

b Ballard et al. 1991.

* *p* < 0.20,

** *p* < 0.05,

# *p* < 0.01.

**Table 2:** Relationship of PCB, MeHg, DDE, HCB, and Pb exposure to DRL IRTs [β (*p*-value)].

	Response bins^a^							
Contaminant (*n*)	1–20	21–40	41–60	61–80	81–100	101–120	121–140	Total (1–140)
Total PCB (167)	−0.071 (0.380)	−0.123 (0.130)	−0.103 (0.206)	−0.195 (0.016)	−0.227 (0.005)	−0.233 (0.004)	−0.235 (0.003)	−0.215 (0.008)^b^
HighCl PCB (167)	−0.185 (0.016)	−0.139 (0.074)	−0.081 (0.300)	−0.158 (0.043)	−0.118 (0.125)	−0.141 (0.069)	−0.139 (0.072)	−0.170 (0.030)^b^
MeHg 1st (151)	−0.200 (0.013)	−0.145 (0.071)	−0.087 (0.277)	−0.099 (0.228)	−0.132 (0.103)	−0.139 (0.086)	−0.209 (0.009)	−0.166 (0.041)^c^^,^^d^
MeHg 2nd (143)	−0.206 (0.013)	−0.182 (0.029)	−0.086 (0.303)	−0.110 (0.190)	−0.152 (0.068)	−0.183 (0.028)	−0.198 (0.017)	−0.184 (0.027)^c^^,^^d^
Composite MeHg (157)	−0.199 (0.012)	−0.164 (0.039)	−0.086 (0.279)	−0.105 (0.185)	−0.145 (0.068)	−0.169 (0.033)	−0.211 (0.007)	−0.178 (0.024)^c^^,^^d^
DDE (167)	0.065 (0.402)	−0.026 (0.730)	0.040 (0.605)	0.012 (0.869)	0.040 (0.604)	−0.006 (0.928)	0.008 (0.914)	0.019 (0.804)
HCB (167)	0.036 (0.635)	−0.053 (0.489)	0.050 (0.517)	0.140 (0.070)	0.107 (0.168)	0.077 (0.319)	0.115 (0.137)	0.091 (0.241)
Postnatal Pb (123)	−0.229 (0.043)	−0.120 (0.281)	−0.143 (0.203)	−0.187 (0.090)	−0.137 (0.218)	−0.194 (0.081)	−0.177 (0.113)	−0.194 (0.081)
Prenatal Pb (179)	−0.065 (0.468)	0.089 (0.324)	0.012 (0.892)	0.112 (0.216)	0.114 (0.206)	0.057 (0.526)	0.030 (0.736)	0.068 (0.450)

β covariate-adjusted standardized beta coefficients; *p*-value, two-tailed significance level; total PCB, sum of all PCB congeners (cord blood); highCl PCB, sum of highly chlorinated PCBs (cord blood); DDE and HCB in cord blood; MeHg 1st and 2nd, maternal hair MeHg 1st and 2nd half of pregnancy (maternal hair).

a Twenty responses in each bin.

b Results unchanged after control for MeHg.

c Results unchanged after control for PCB.

d Results unchanged after removal of subject with MeHg level > 2.0.

**Table 3:** Relationship of PCBs, MeHg, DDE, HCB, and Pb exposure to money earned during the DRL task.

Contaminant	β	*p*-Value
Total PCB	−0.208	< 0.010
HighCl PCB	−0.076	0.338
MeHg 1st	−0.194	< 0.026
MeHg 2nd	−0.203	< 0.027
DDE	0.049	0.521
HCB	0.096	0.212
Postnatal Pb	−0.195	< 0.047
Prenatal Pb	0.035	0.641

**Table 4:** Relationships between contaminants (PCBs and MeHg) and DRL performance with and without simultaneous control of each other.

Contaminant	No.	Beta	*p*-Value
Total PCB	167	−0.215	0.008
Total PCB in MeHg subsample	145	−0.218	0.012
Total PCB with MeHg controlled 145	145	−0.198	0.022
High chlorine PCB	167	−0.170	0.030
High chlorine PCB in MeHg subsample	145	−0.214	0.011
High chlorine PCB with MeHg controlled	145	−0.192	0.020
MeHg	157	−0.178	0.024
MeHg in PCB subsample	145	−0.204	0.013
MeHg with PCB controlled	145	−0.179	0.029
